# Supplementary material for: Immune-suppression by OsHV-1 viral infection causes fatal bacteraemia in Pacific oysters
Source: Nat Commun. 2018 Oct 11;9:4215. doi: 10.1038/s41467-018-06659-3 (PMC6182001; doi:10.1038/s41467-018-06659-3)
Supplement: Supplementary file 1 — Supplementary Information [file 41467_2018_6659_MOESM1_ESM.pdf]

Supplementary Information

Immune-suppression by OsHV-1 viral infection causes fatal bacteremia in Pacific oysters

de Lorgeril et al.

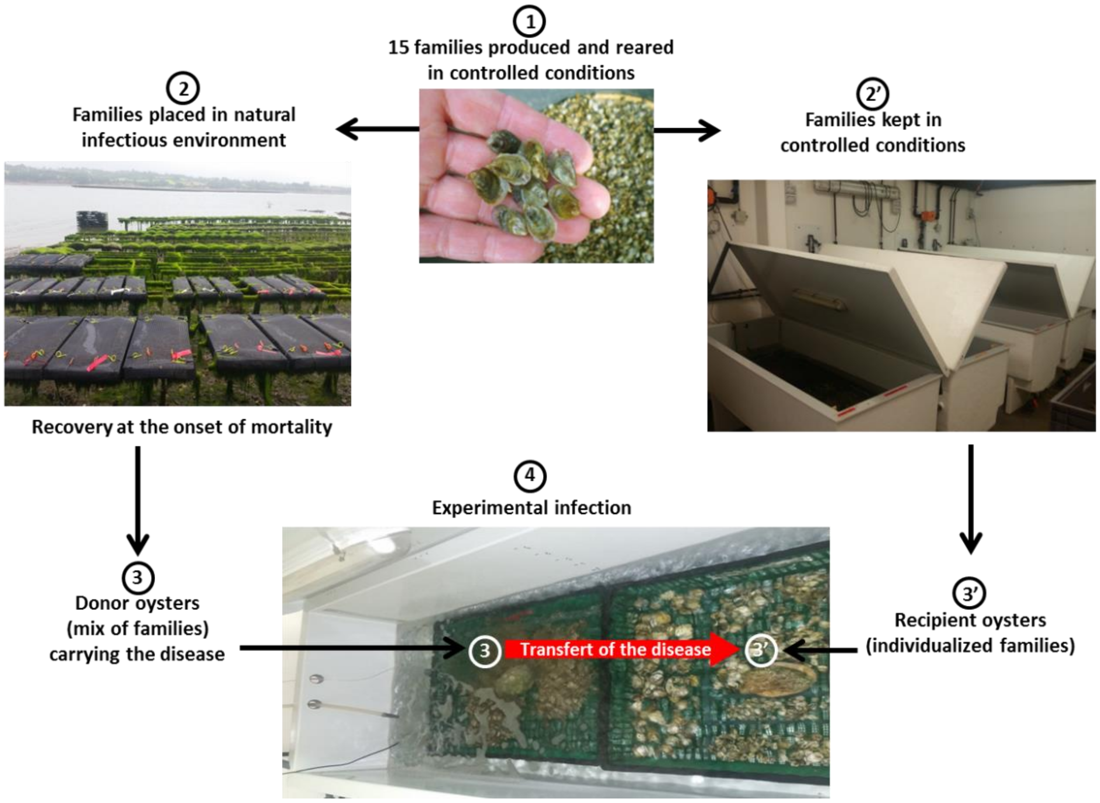

Supplementary Figure 1: Description of the 'natural' experimental infection. The protocol was derived from those used in previous studies [1,2]. The oysters were from the families produced and maintained in controlled and biosecured conditions until experimental infection (1). Half of the oysters were placed in a farming area during an infectious period (2) and became the donor oysters carrying the disease (3), while the other half remained in controlled and biosecured conditions (2') and were used as recipient oysters (3'). As soon as mortalities occurred among the donor oysters placed in the natural environment, they were transferred back to the laboratory. During the infection, the transfer of disease was performed by placing recipient oysters (*n*=1000) from each family in a tank with a mixture of donor oysters (*n*=1000) from the 15 families carrying the disease (4). A control was created by placing each of the 15 families in contact with a mixture of oysters that were not exposed to the infectious environment. During the 'natural' experimental infection, 3 triplicates of 10 oysters were sampled from each tank and each time (0, 6 h, 12 h, 24 h, 48 h, 60 h and 72 h) of the kinetics for further molecular analysis.

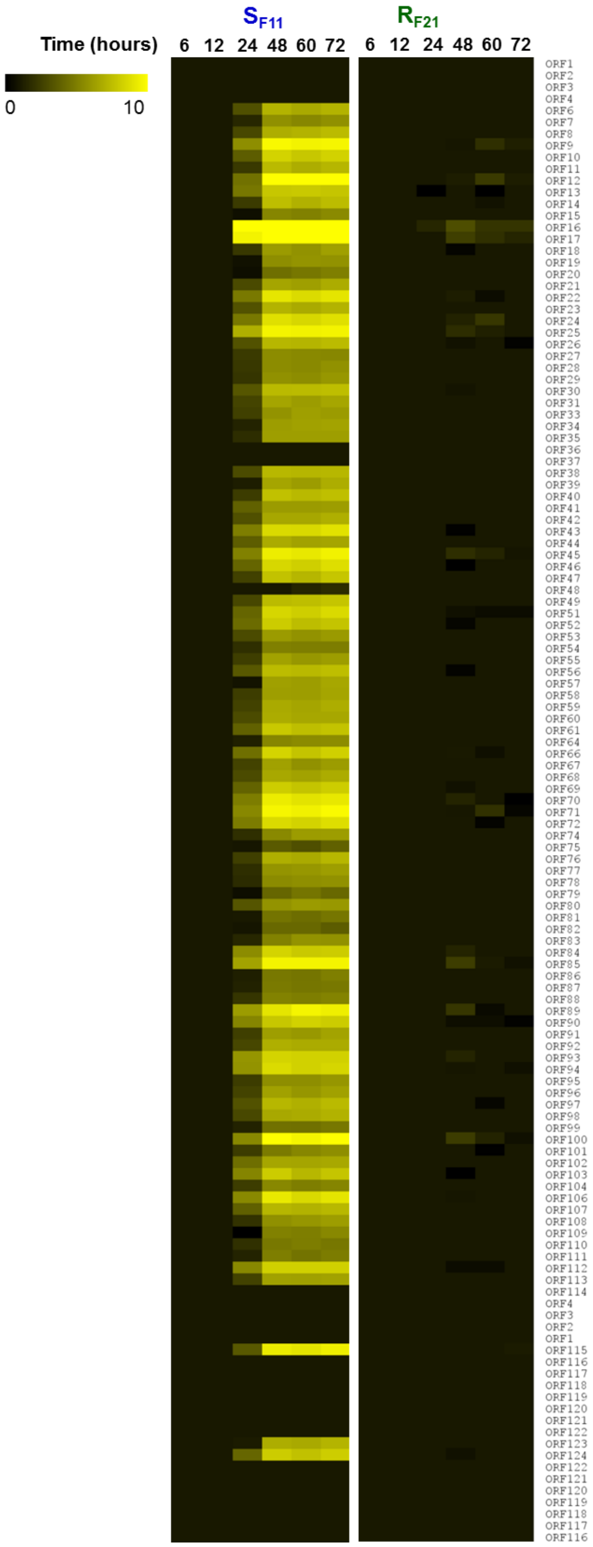

Supplementary Figure 2: Time-course of OsHV-1 ORF expression during the 'natural' experimental infection in the susceptible S_{F11} and in the resistant R_{F21} oysters. The fold changes in OsHV-1 ORF expression were calculated between each time point of the kinetics and the T0. Analyses were conducted with RNA-seq data through mapping against the OsHV-1 genome [3]. The intensity of the colour indicates the magnitude of the differential expression (log2 fold change). The heat map was constructed with Multiple Array Viewer software. ORF numbers from the reference genotype are indicated.

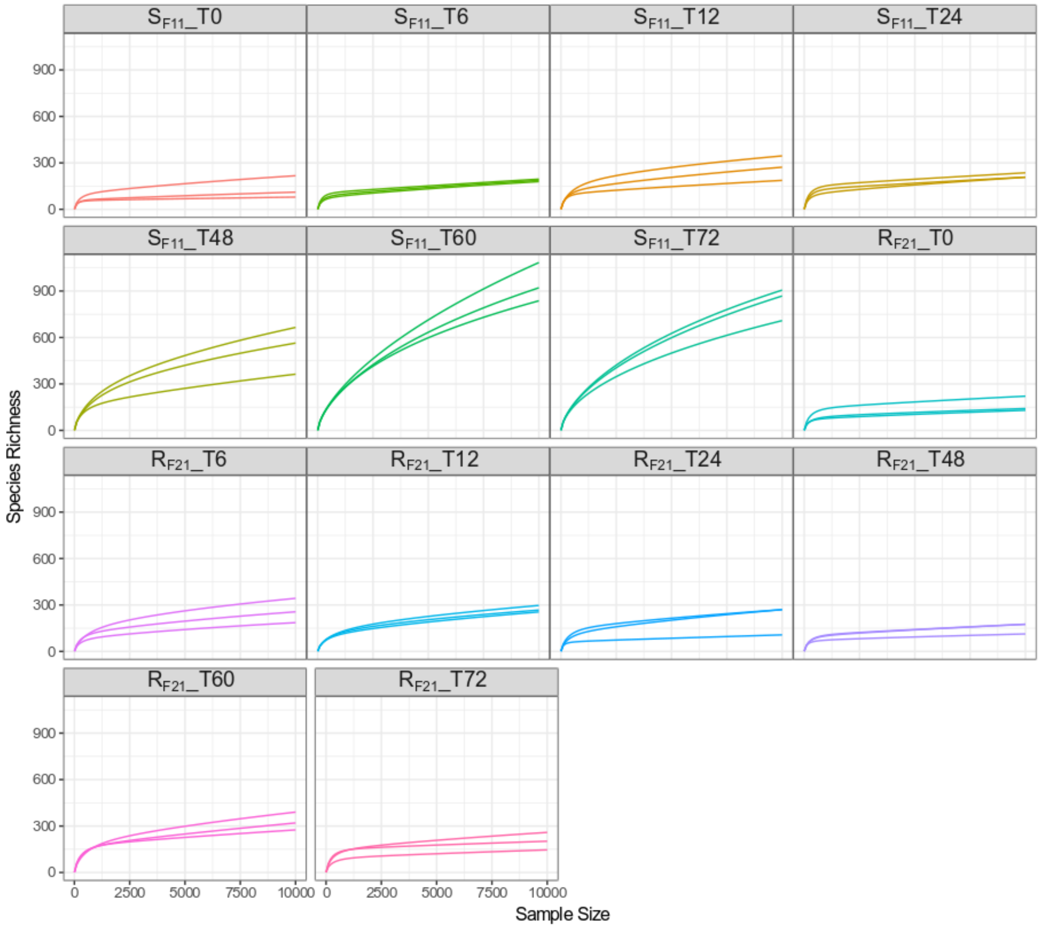

Supplementary Figure 3: Richness rarefaction curves of the sub-sampled 16S rDNA dataset (10000 reads per sample) for the susceptible S_{F11} and the resistant R_{F21} oysters showing the number of OTUs as a function of the number of sequences analysed (sample size). T0, T6, T12, T24, T48, T60 and T72 correspond to the different sampling times (in hours) during the kinetics of the 'natural' experimental infections. Triplicate results are shown for each time point.

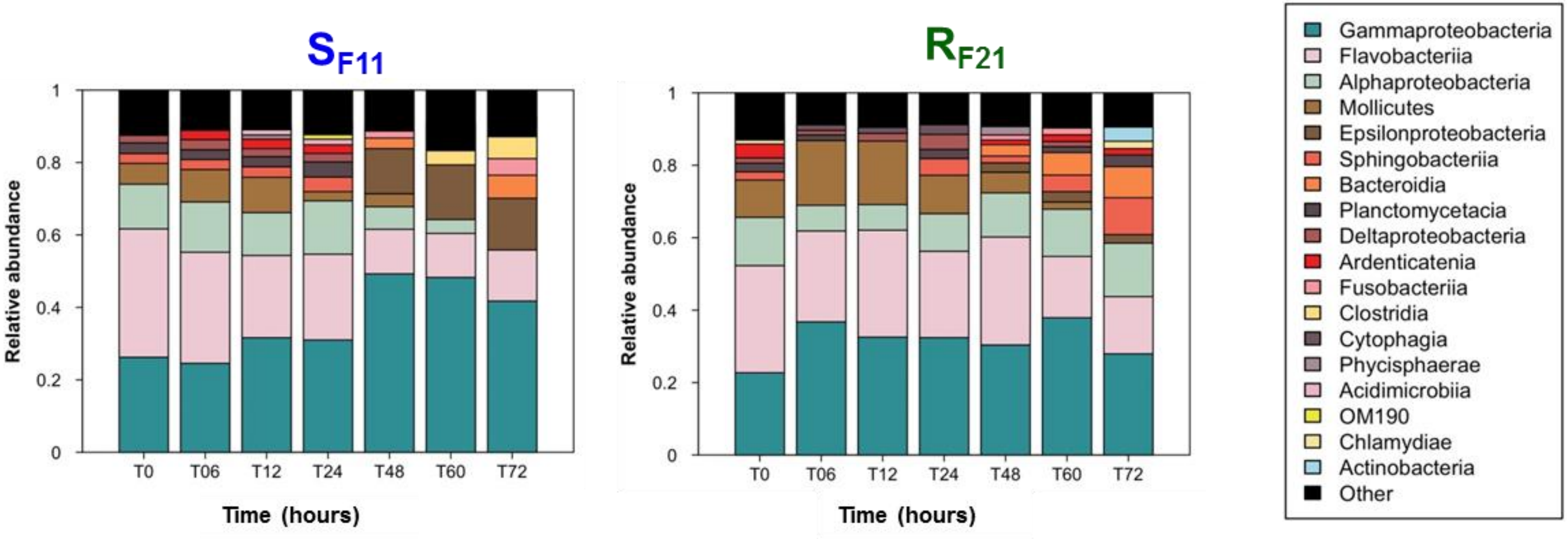

Supplementary Figure 4: Relative proportions of bacteria (class level) for the susceptible S_{F11} and the resistant R_{F21} oysters. T0, T06, T12, T24, T48, T60 and T72 correspond to the different sampling times (in hours) during the kinetics of the 'natural' experimental infections. The average percentages of reads obtained for the three distinct pools of 10 oysters are shown at each time.

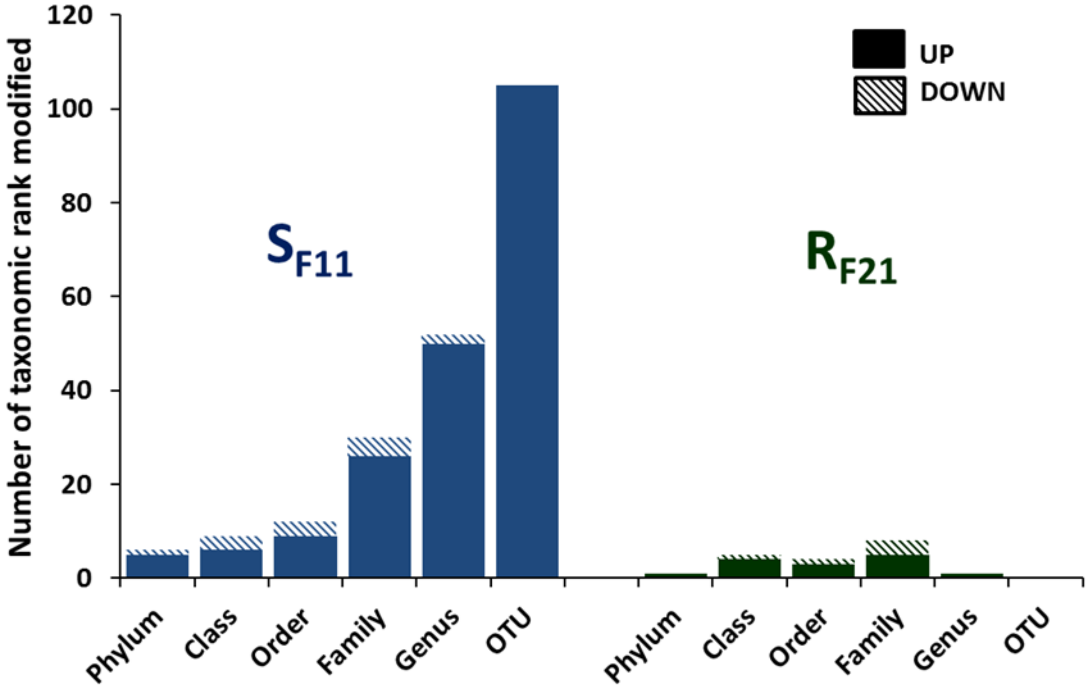

Supplementary Figure 5: Microbiota modifications analysed by 16S rDNA metabarcoding in the susceptible S_{F11} and in the resistant R_{F21} oysters during the 'natural' experimental infection. Significant modifications (up and down; DESeq2, $p < 0.05$) between the initial and the final time point of the kinetics were much more important at each taxonomic rank (from the phylum to the OTU level) for susceptible S_{F11} compared with resistant R_{F21} oysters.

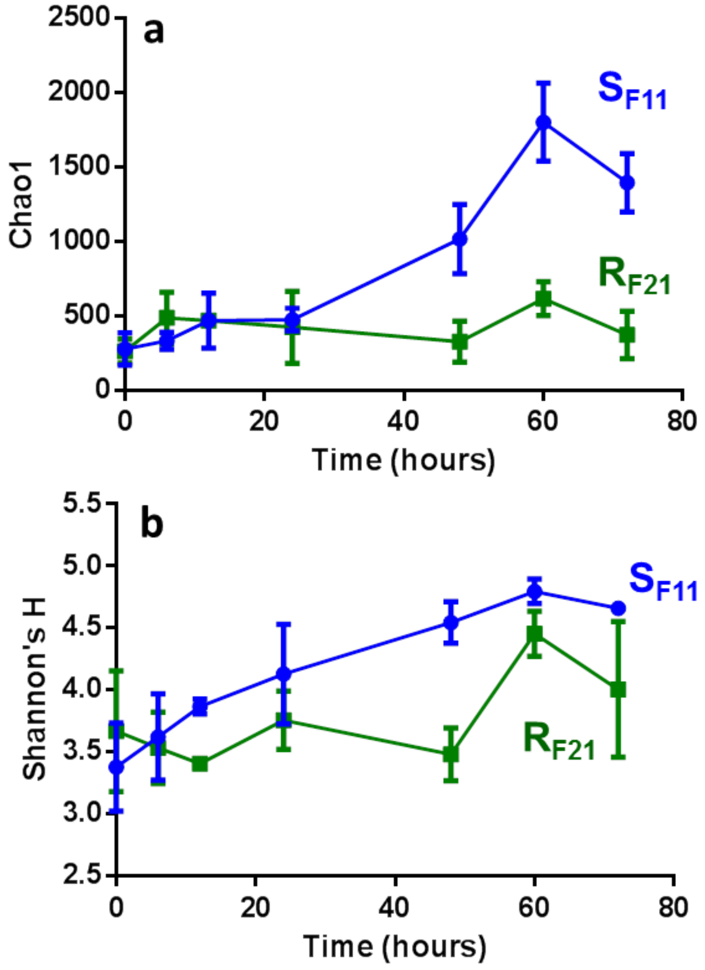

Supplementary Figure 6: Temporal dynamics of alpha diversity during the 'natural' experimental infection. Chao1 **(a)** and Shannon's H index **(b)** for the susceptible S_{F11} (in blue) and the resistant R_{F21} (in green) oysters. The data (obtained for the three distinct pools of 10 oysters) are presented as mean ± S.D.

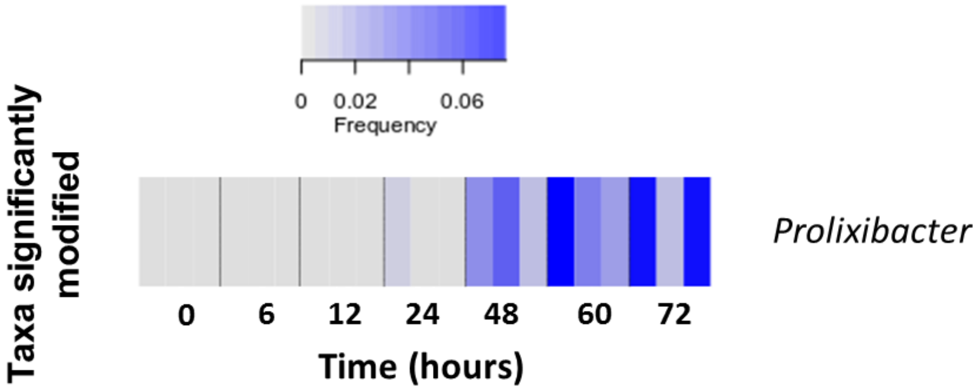

Supplementary Figure 7: Heatmap of the only bacterial genus (*prolixibacter*) that is significantly modified in the resistant R_{F21} oysters during the 'natural' experimental infection. The intensity level of blue represents the relative abundance of this genus (frequency) at different time points of the kinetics. At each time, the analysis was performed on 3 distinct pools of 10 oysters.

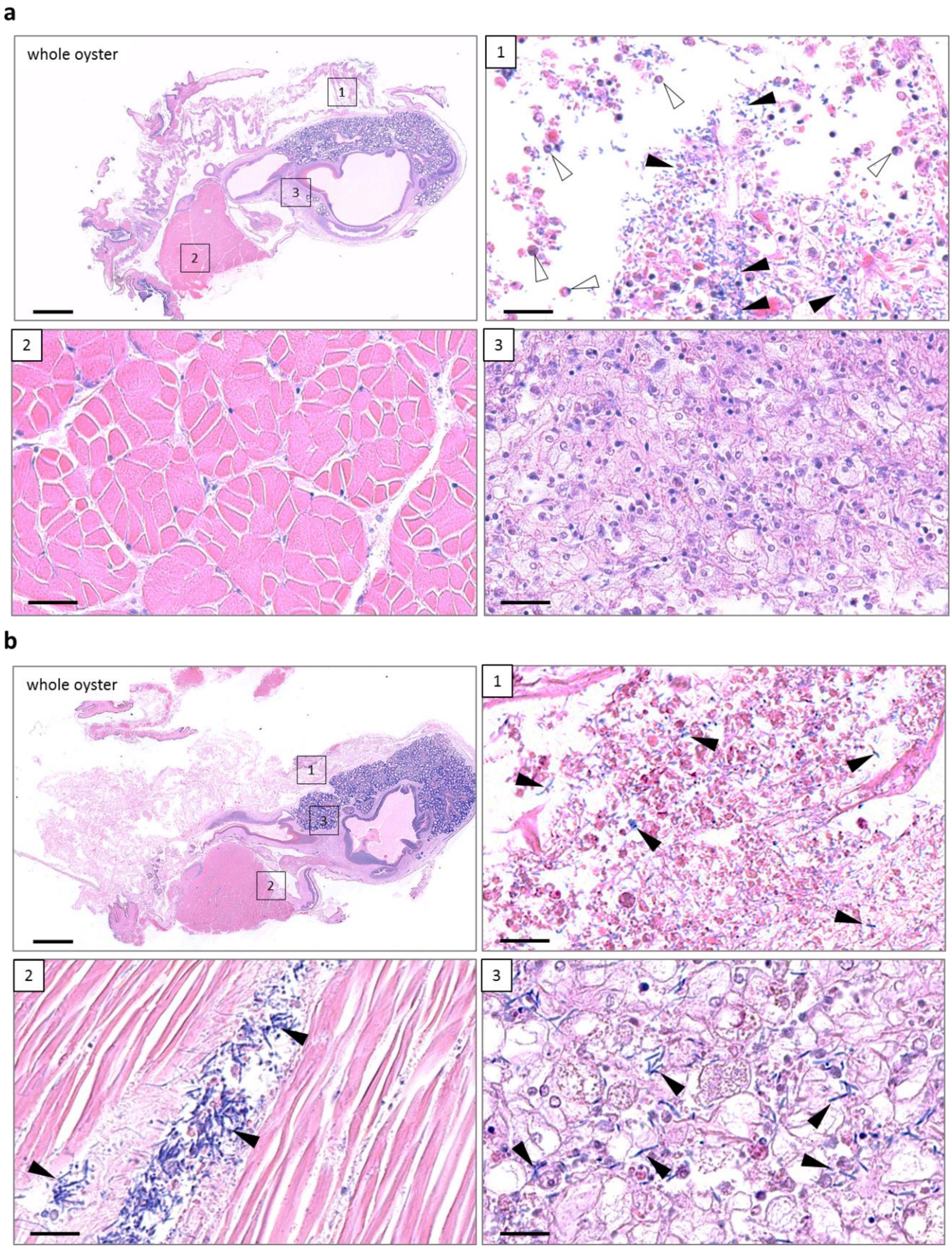
a
whole oyster
1
2
3
1
2
3
b
whole oyster
1
2
3
1
2
3

c

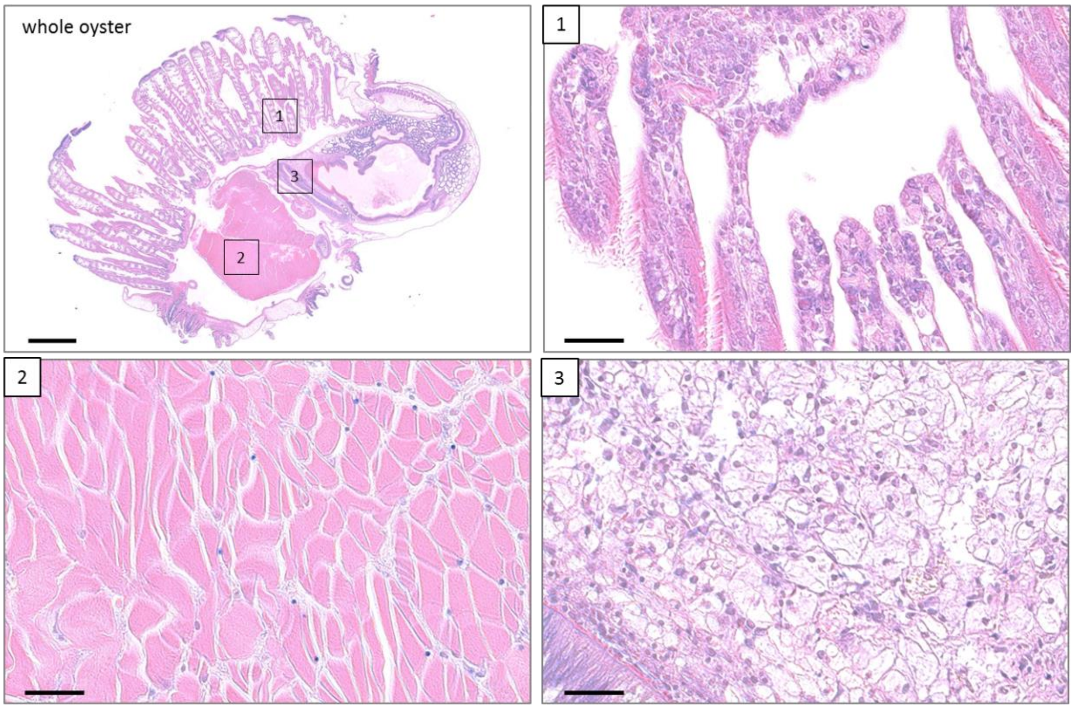

d

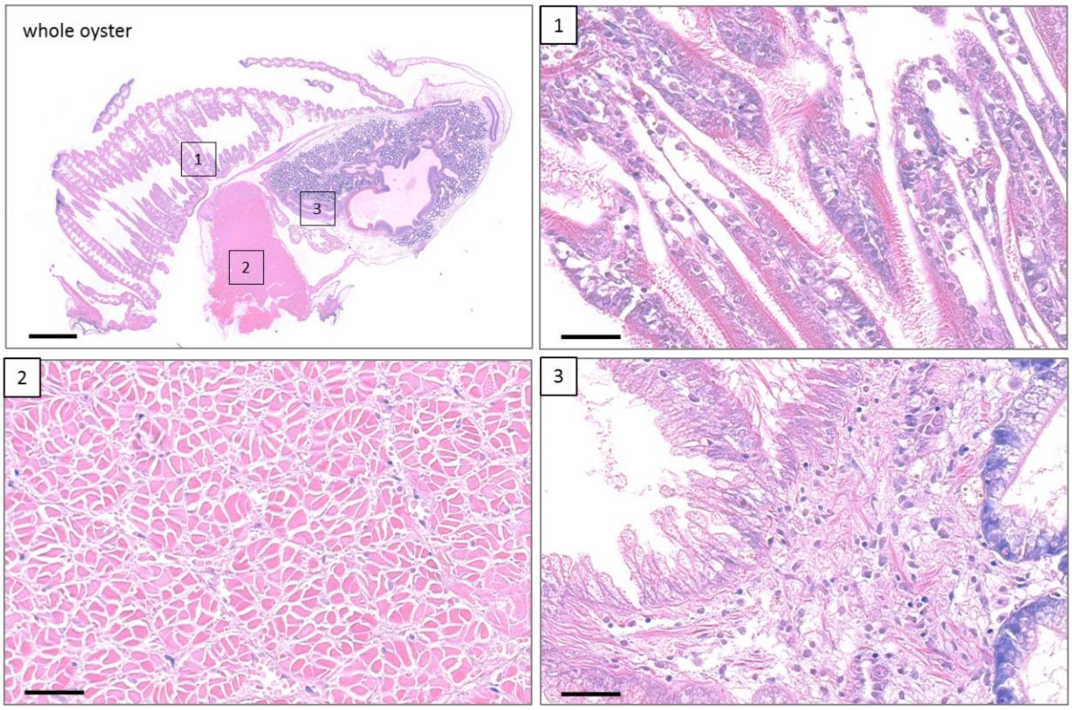

e

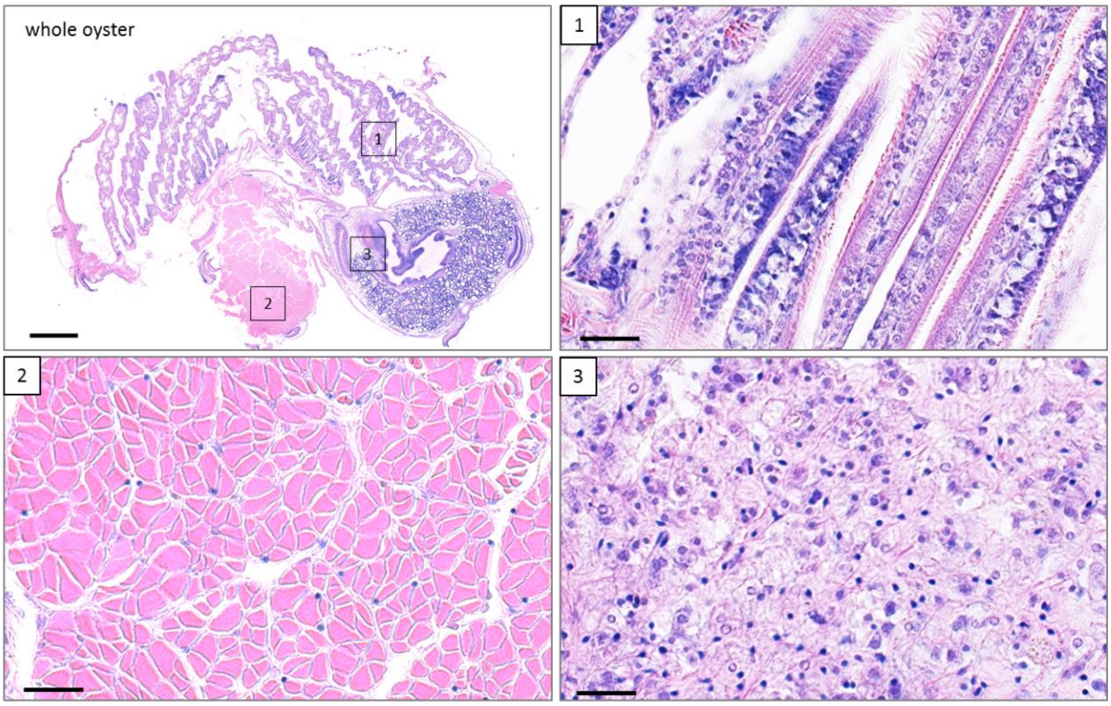

f

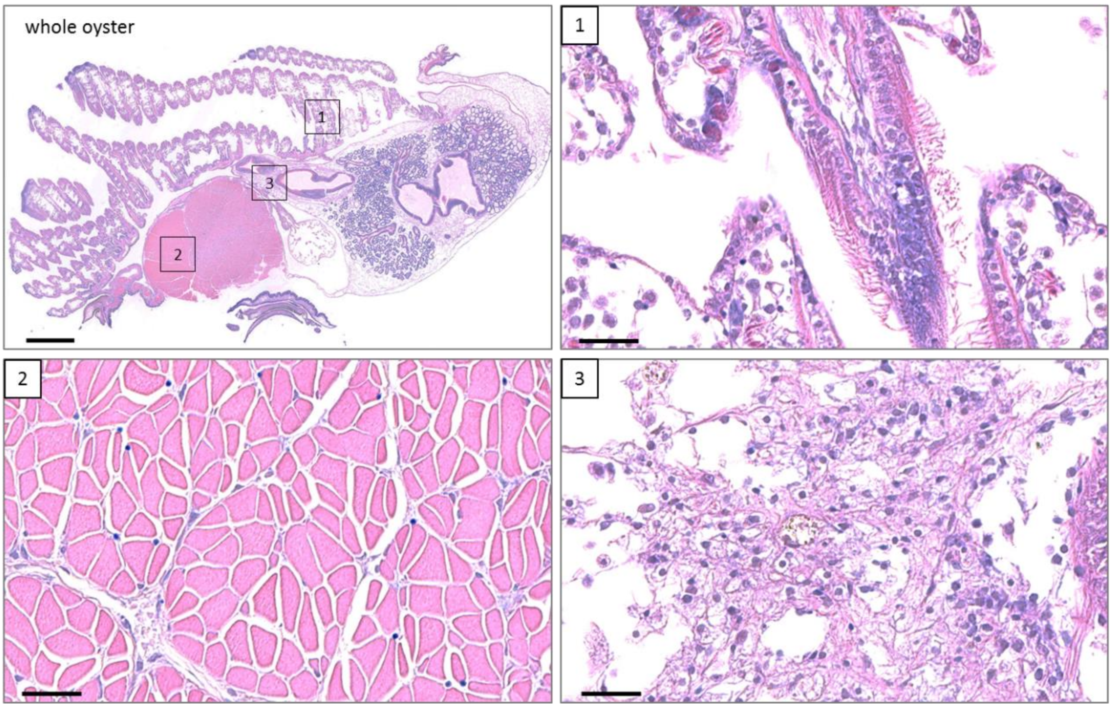

Supplementary Figure 8: Histological tissue analysis by Giemsa staining showing bacteraemia in the susceptible S_{F11} but not in the resistant R_{F21} oysters during the 'natural' experimental infection. Giemsa staining was performed on paraffin wax-embedded sections of animals that were sampled at different time points to visualize tissue colonised by bacteria. Oyster tissues and cells were coloured in shades of pink to purple, and most bacteria were coloured in deep blue by Giemsa staining. **(a)** At 54 h after the beginning of the experiment, bacteria started to accumulate at the interfaces of the gills and the mantle epithelia in S_{F11} animals (filled arrowheads). Rounded cells reminiscent of typical haemocytes were observed both in gill tissues and outside any tissues associated with bacteria (open arrowheads). The gills and mantle epithelia appeared damaged at many different sites, with altered tissue integrity. **(b)** At 78 h after the beginning of the experiment, gill tissues appeared massively degraded (see whole oyster and gill images), and bacteria were found in most of the tissues of the S_{F11} animals, for example, the adductor muscle or the interstitial tissue near the digestive tract. No bacteria or tissue damage were observed in animal sections of S_{F11} **(c)** or R_{F21} **(d)** at the beginning of the infection or at any time points for the R_{F21} sections **(e, f)**. In **(a-f)** panels, (1) gill, (2) muscle and (3) interstitial tissues are magnified (scale bars = 1mm for whole oyster panel and = 20µM for 1, 2 and 3panels).

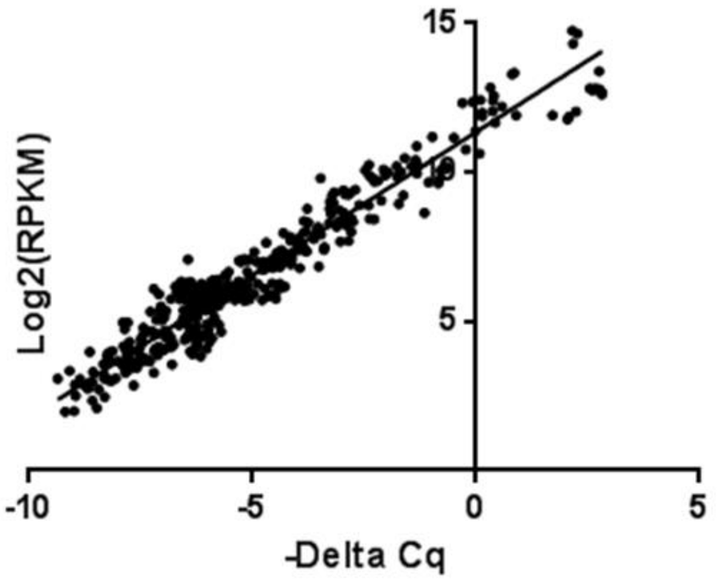

Supplementary Figure 9: Validation of the RNA-seq data by RT-qPCR. Thirty genes with contrasting expression levels were selected (the list of primers and PCR efficiencies are shown in Supplementary Table 2), and their relative expression levels were quantified by RT-qPCR using the same RNA used for the RNA-seq approach. These results, expressed as -Delta Cq, were plotted against the log2 (RPKM) obtained by the RNA-seq approach. The regression line ($y = 0.9595x + 11.36$) displaying r^2 of 0.936 indicates a high level of correlation between the two methods.

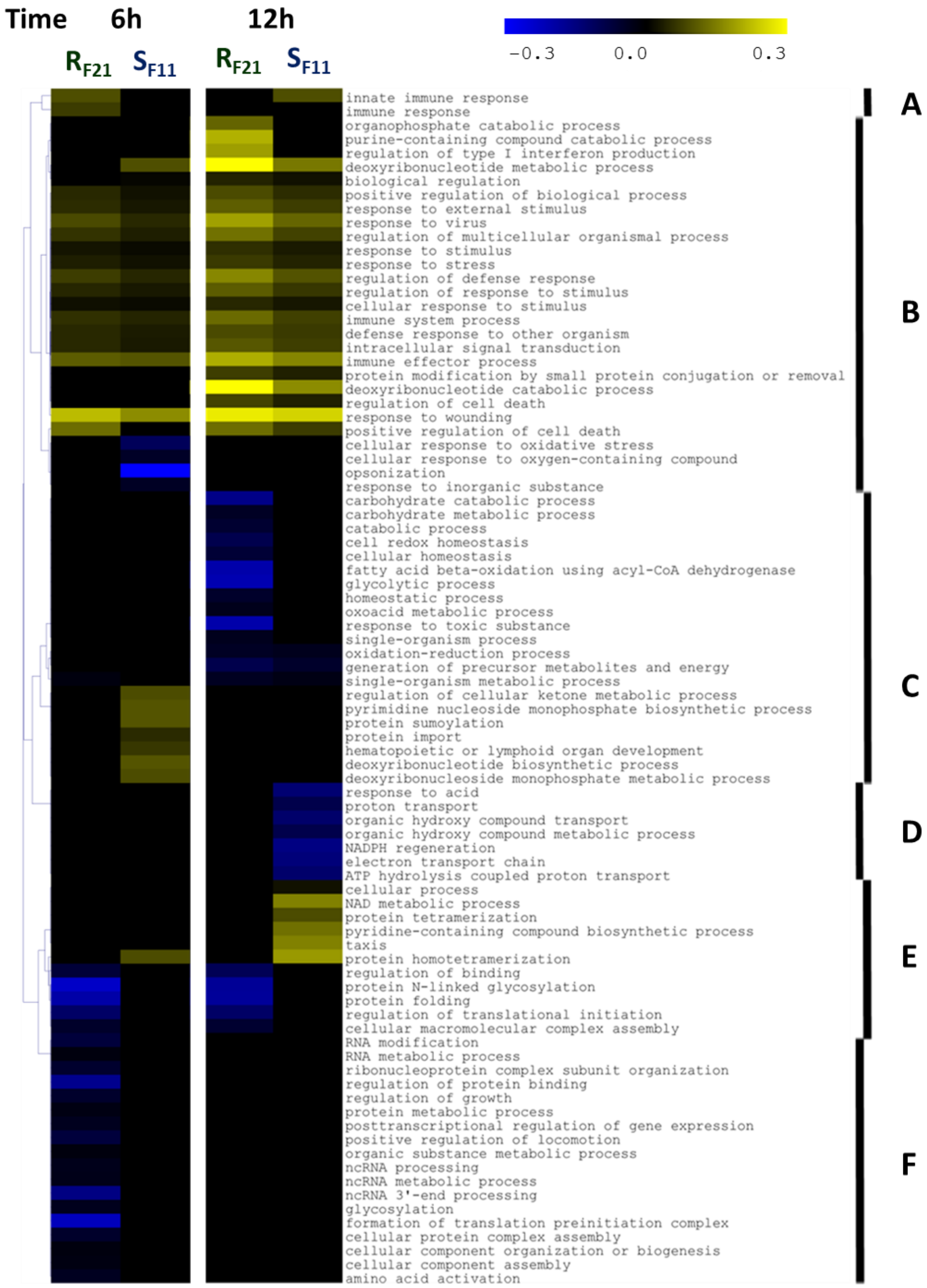

Supplementary Figure 10: Heat map of enriched gene ontology categories for the susceptible S_{F11} and for the resistant R_{F21} oysters at 6 and 12 hours after the beginning of the 'natural' experimental infection. The intensity of the enrichment is expressed as the ratio between the number of genes that were significantly up (yellow heat) or down (blue heat) regulated in the category/total number of genes in the category. If the intensity was equal to zero, the enrichment was not significant for the corresponding condition (black heat). GO categories were clustered (A to F) according to the Pearson correlation (black filled bar).

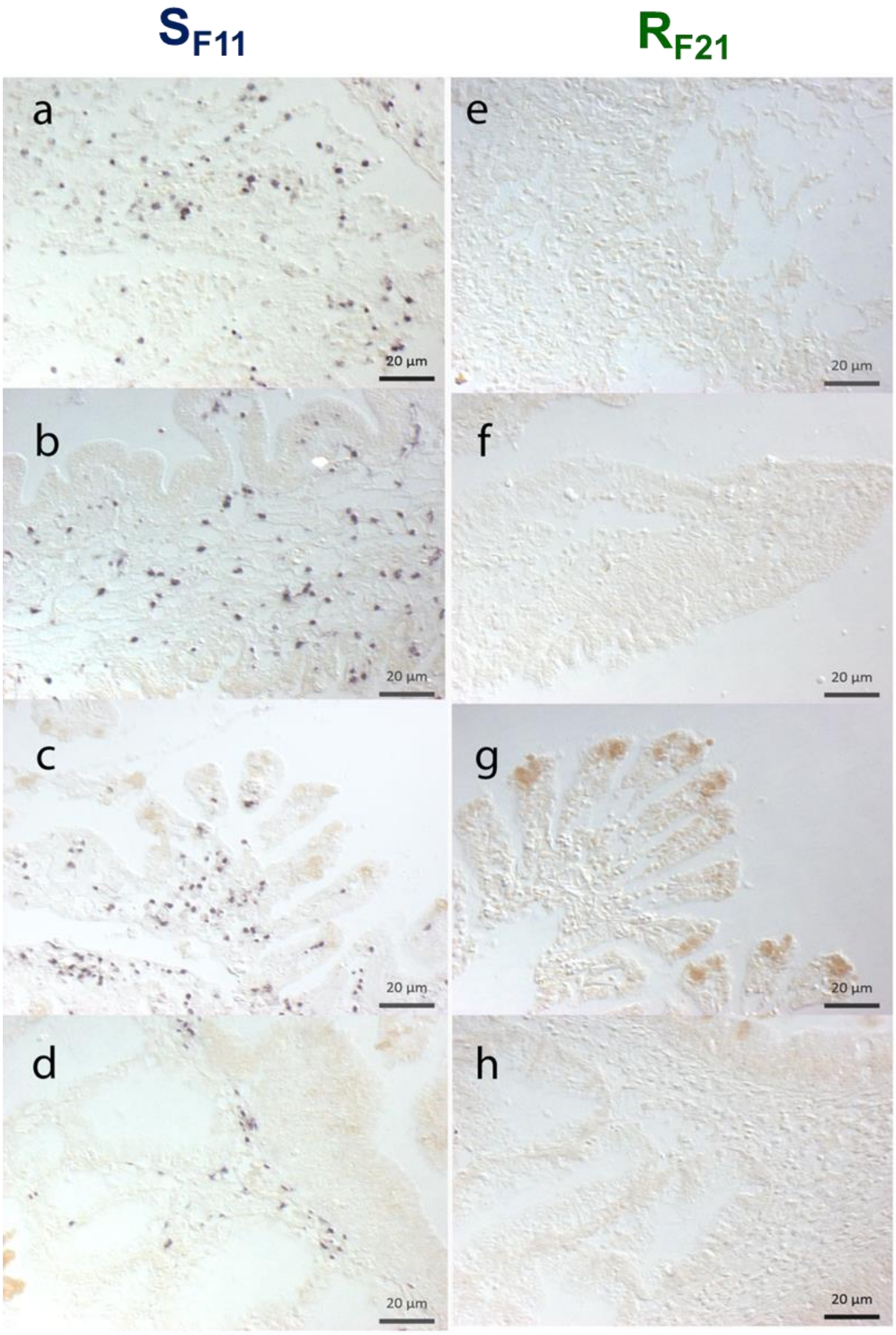

Supplementary Figure 11: ***In situ*** **localization of virus-infected cells in the suceptible S_{F11} (a-d) and in the resistant R_{F21} (e-h) oysters at 54 h after the beginning of the 'natural' experimental infection.** OsHV-1 was detected by *in situ* hybridization. Paraffin wax-embedded sections of oysters fixed 54 h after infection were hybridized with a specific probe labelled with digoxigenin and revealed using alkaline phosphatase-conjugated antibodies and NBT/BCIP (dark blue precipitate). Labelling was observed only in S_{F11} tissues (**a,e**: heart; **b,f**: mantle; **c,g**: gills; **d,h**: digestive gland and connective tissue). Scale bars = 20µm.

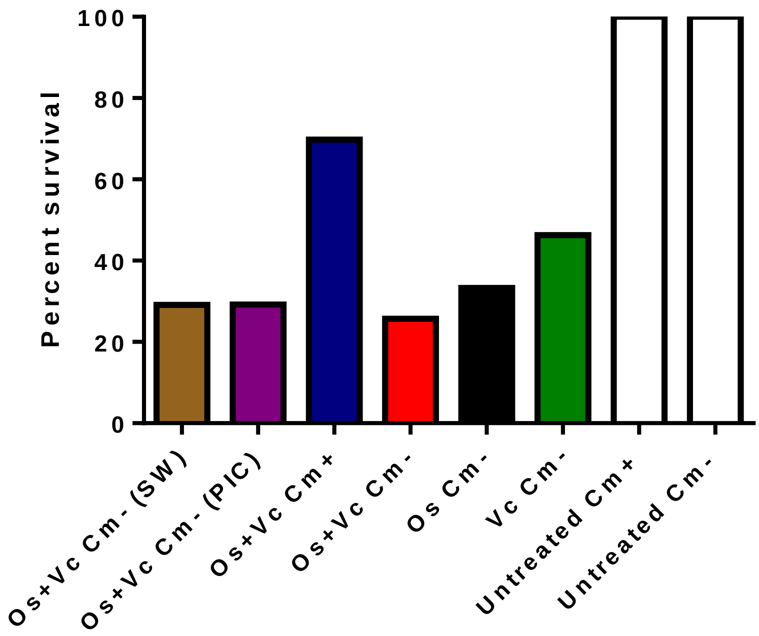

Supplementary Figure 12: Percent survival of donor oysters (genetically diversified) at the end of the rationalized experimental infections (72 h) by OsHV-1 and/or *V. crassostreae*. Donor oysters (*n*=100) were injected with either 3.88×10^{8} genomic units of OsHV-1 (Os) or with 5×10^{7} cfu of *V. crassostreae* (Vc). Recipient oysters (*n*=100) were injected with poly(I:C) (PIC) or sterile seawater (SW) before exposure to both Os and Vc donors (Os+Vc). Recipients were exposed to both Os and Vc donors (Os+Vc) in the presence (Cm+) or absence (Cm-) of chloramphenicol in the tanks. Recipients were exposed to Os or Vc donors in the absence of chloramphenicol (Cm-). Recipients were exposed to untreated donors in the presence (Cm+) or absence (Cm-) of chloramphenicol. Histograms represent the percent survival of donors in these different experiments.

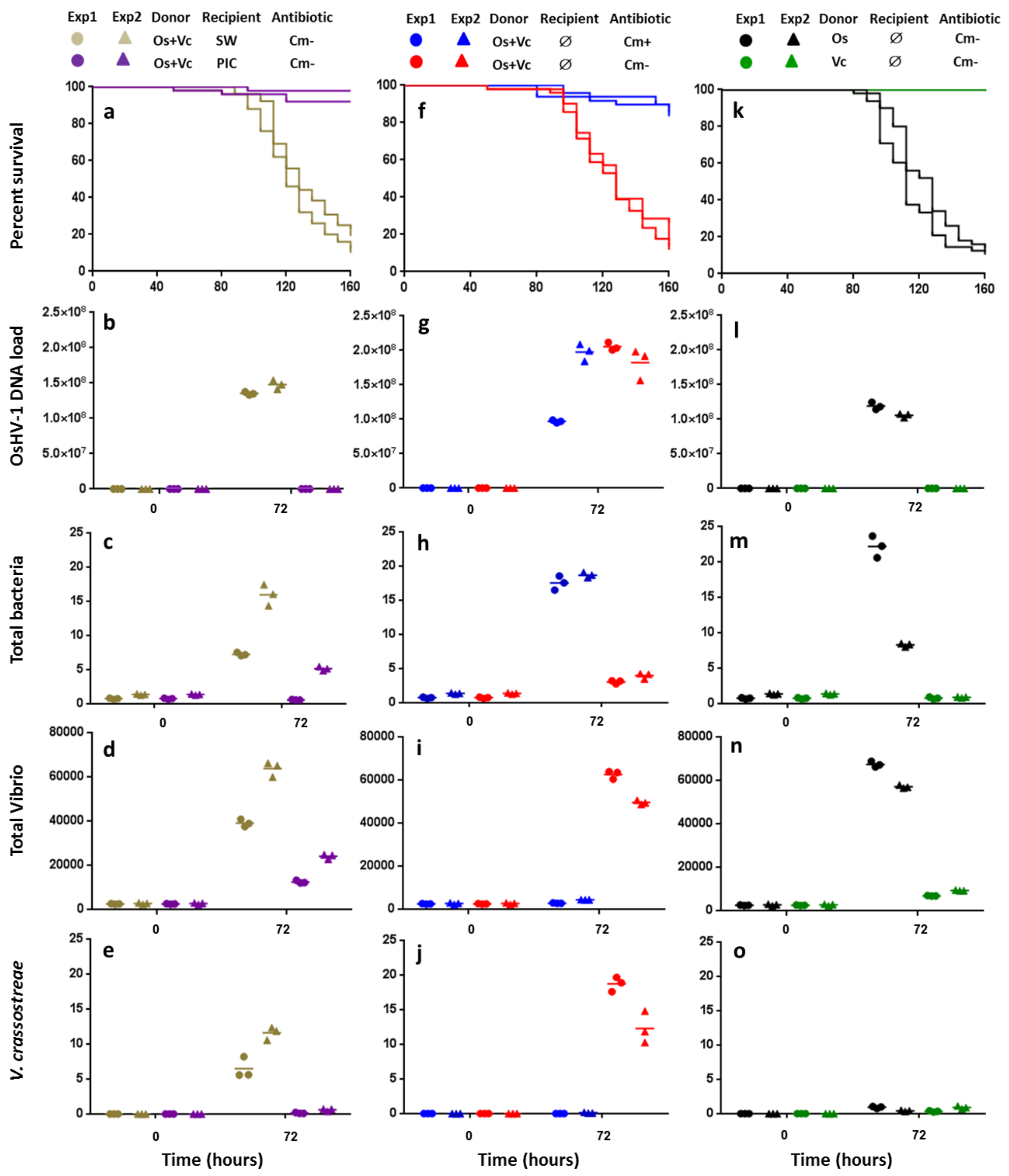

Supplementary Figure 13: Rationalized infection experiments using susceptible oysters from the H12 biparental family. Two additional rationalized infections (Exp1 and Exp2) by OsHV-1 and/or *V. crassostreae* were performed: oyster donors (*n*=100) were injected with 3.88×10^{8} genomic units of OsHV-1 (Os) or 5×10^{7} cfu *V. crassostreae* (Vc). **(a-e)** Recipient oysters (*n*=100) were injected with poly(I:C) (PIC) or sterile seawater (SW) before exposure to both Os and Vc donors (Os+Vc). **(f-j)** Recipients oysters (*n*=100) were exposed to both Os and Vc donors (Os+Vc) in the presence (Cm+) or absence (Cm-) of chloramphenicol in the tanks. **(k-o)** oysters (*n*=100) were exposed to Os or Vc donors (Os+Vc). Mortalities and pathogen loads of recipient oysters were monitored during disease development. The OsHV-1 DNA load (viral genomic units per ng of total oyster DNA) and relative or absolute quantification of total bacteria, total Vibrio and *V. crassostreae* abundance were measured by

qPCR. Injection of poly(I:C), as opposed to SW, was sufficient to completely block OsHV-1 replication (**b**), bacterial colonization (**c-e**) and death of recipient oysters (**a**). Moreover, antibiotic treatment significantly reduced the load of vibrios (**i**, pairwise *t.test* at T72h; d.f.=10; $p < 0.0001$), including *V. crassostreae* (**j**, pairwise *t.test* at T72h; d.f.=10; $p < 0.0001$) and oyster mortality (**f**, Mantel-Cox log-rank test, $p < 0.0001$) without affecting OsHV-1 replication (**g**). When only one pathogen was injected into donor oysters (**k-o**), recipient oyster mortality was observed only when they were exposed to virus-injected donors (**k**). In this condition, viral replication (**l**) was accompanied by an increase both in the total bacterial load (**m**, pairwise *t.test* at T72h; d.f.=10; $p < 0.001$) and in the total vibrio load (**n**, pairwise *t.test* at T72h; d.f.=10; $p < 0.0001$). Nevertheless, *V. crassostreae*, which was not included in this last experimental infection set-up, was not detected in oyster flesh (**o**). No mortality was observed, and no OsHV-1 DNA was detected in recipient oysters when untreated donors were used as a control.

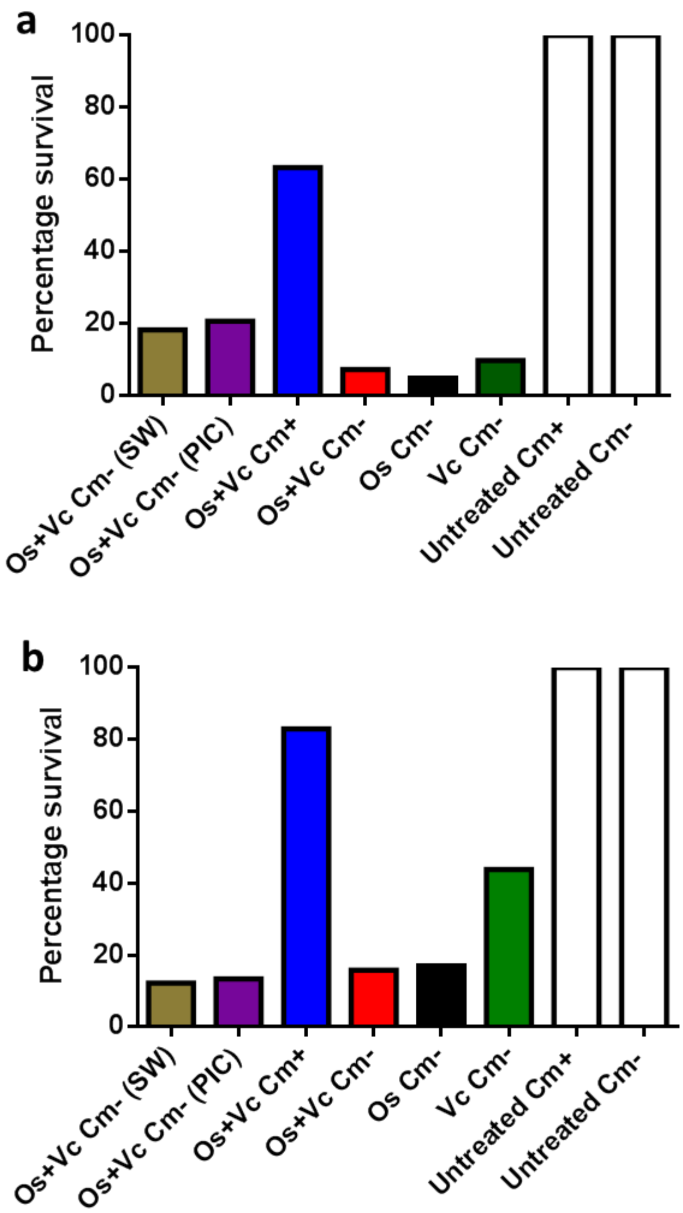

Supplementary Figure 14: Percent survival of donor oysters (H12 biparental family) at the end of two independent rationalized experimental infections (72 h) by OsHV-1 and/or *V. crassostreae*. Panel **(a)** and **(b)** represent the results of experimentation 1 (Exp 1) and 2 (Exp 2), respectively (Supplementary Figure 13). Donor oysters (*n*=100) were injected with either 3.88×10^8 genomic units of OsHV-1 (Os) or with 5×10^7 cfu of *V. crassostreae* (Vc). Recipient oysters (*n*=100) were injected with poly(I:C) (PIC) or sterile seawater (SW) before exposition to both Os and Vc donors (Os+Vc). Recipients (*n*=100) were exposed to both Os and Vc donors (Os+Vc) in the presence (Cm+) or absence (Cm-) of chloramphenicol in the tanks. Recipients were exposed to Os or Vc donors in the absence of chloramphenicol (Cm-). Recipients (*n*=100) were exposed to untreated donors in the presence (Cm+) or absence (Cm-) of chloramphenicol. Histograms represent the percent survival of donors in these different experiments.

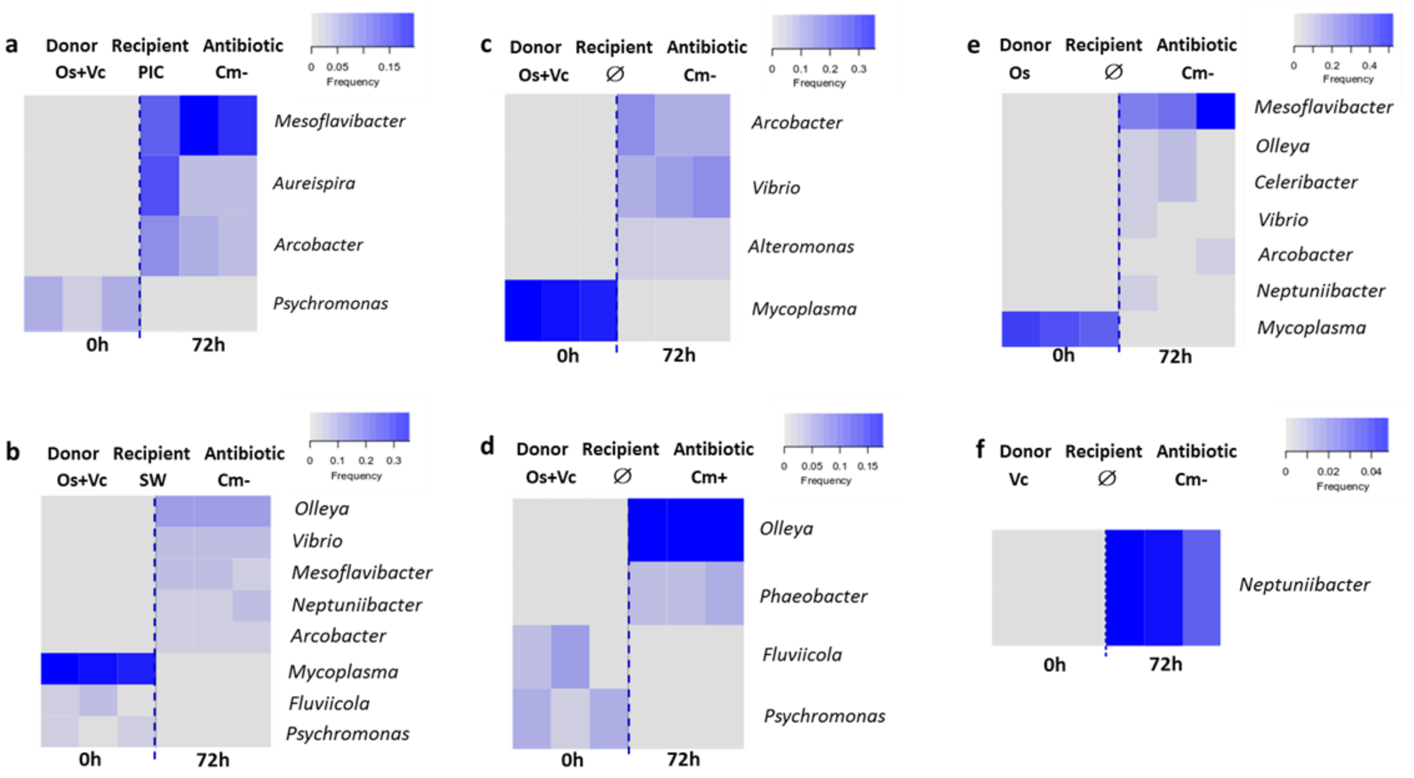

Supplementary Figure 15: Heatmaps of bacterial communities that are significantly modified during the rationalized experimental infections by OsHV-1 and/or *V. crassostreae*. Donor and recipient 'pathogen-free' oysters were used for these experiments. Donor oysters were injected with either 3.88×10^8 genomic units of OsHV-1 (Os) or 5×10^7 cfu of *V. crassostreae* (Vc). Recipient oysters were injected with **(a)** polyI:C (PIC) or **(b)** sterile seawater (SW) before exposition to both Os and Vc donors (Os+Vc). Recipient oysters were exposed to both Os and Vc donors in the **(c)** absence (Cm-) or **(d)** presence (Cm+) of chloramphenicol in the tanks. Recipient oysters were exposed to Os donors **(e)**. Recipient oysters were exposed to non-injected donors (**f**, control). Analyses were performed at the genus level. Only genera with a relative proportion superior to 4% in one sample are shown. The intensity level of blue represents the relative abundance of genera. No significant modification of the bacterial taxa was observed for the recipient oysters exposed to Vc donors. At each time, the analysis was performed on 3 distinct pools of 10 oysters.

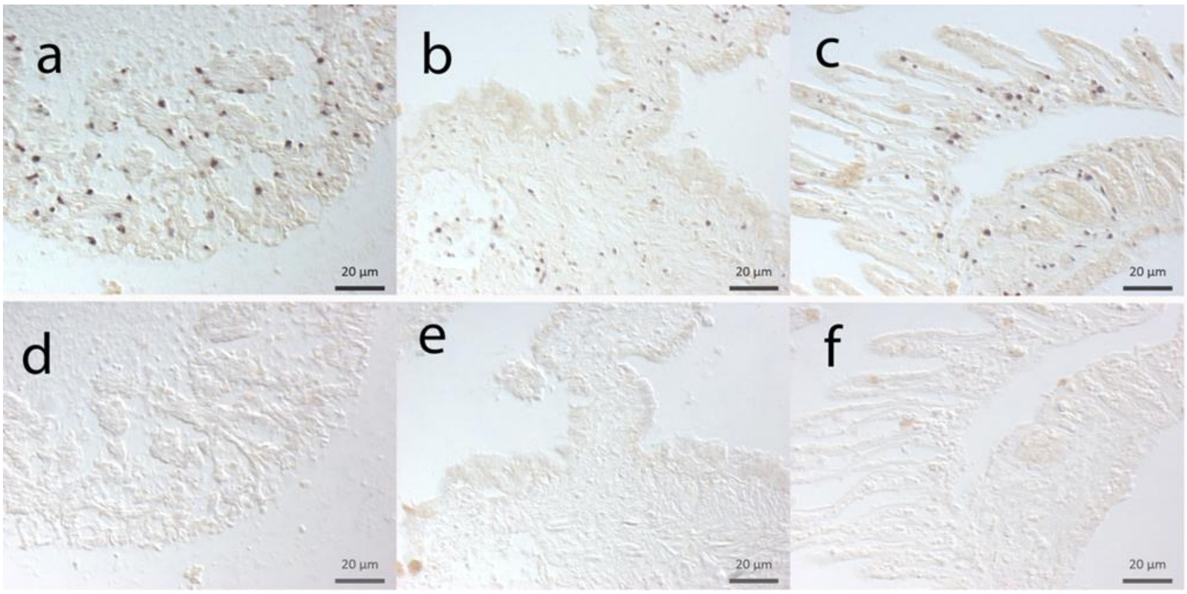

Supplementary Figure 16: Controls for *in situ* localization of virus-infected-cells. OsHV-1 was detected in the susceptible S_{F11} oysters by *in situ* hybridization. Paraffin wax-embedded consecutive sections of oysters that were fixed 54 h after infection were hybridized with **(a-c)** a virus-specific or **(d-f)** a GFP-specific probe as a control. The probes were labelled with digoxigenin and revealed using alkaline phosphatase-conjugated antibodies and NBT/BCIP (dark blue precipitate). In all tissues, labelling was observed only with the virus-specific probe (**a,d**: heart; **b,e**: mantle; **c,f**: gills). Scale bars = 20µm.

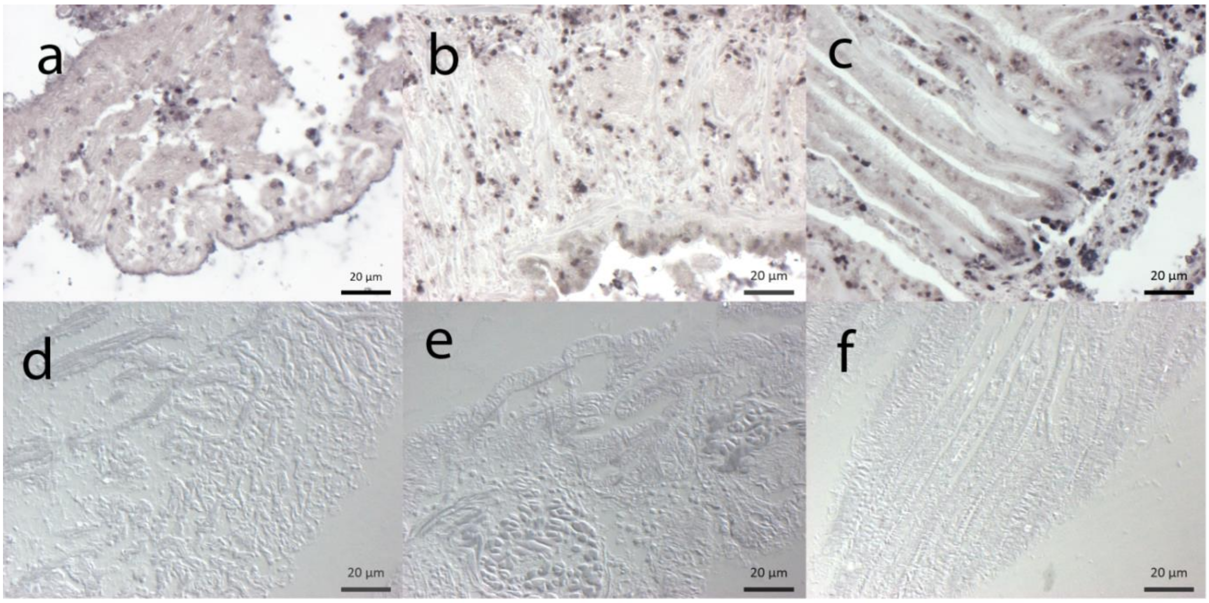

Supplementary Figure 17: Controls for *Cg*-EcSOD immuno-localization. SOD was detected in susceptible oysters (S_{F11}) by immunostaining. Paraffin wax-embedded sections of oysters were incubated **(a-c)** with an antibody specific to the SOD haemocytic protein or **(d-f)** without any primary antibody as a control. Immunostaining was revealed using alkaline phosphatase-conjugated secondary antibodies and NBT/BCIP (dark blue precipitate). In all tissues, labelling was observed only in cells interpreted as haemocytes with the SOD antibody (**a,d**: heart; **b,e**: mantle; **c,f**: gills). Scale bars = 20µm.

Supplementary Table 1 : Oysters broodstock origin

**Family**	**broodstock origin**	**Site**	Geographical coordinate
**F1**	Natural environment – farming area	Atlantic	Logonna Daoulas (lat 48.335263 – long -4.317922)
**F2**	Natural environment – farming area	Atlantic	Logonna Daoulas (lat 48.335263 - long - 4.317922)
**F9**	Natural environment – farming area	Atlantic	Logonna Daoulas (lat 48.335263 - long - 4.317922)
**F11**	Natural environment – non farming area	Atlantic	Dellec (lat 48.353970, long - 4.566123)
**F14**	Natural environment – non farming area	Atlantic	Dellec (lat 48.353970, long - 4.566123)
**F15**	Natural environment – non farming area	Atlantic	Dellec (lat 48.353970, long - 4.566123)
**F21**	Breeding program	Atlantic	Charente Maritime- La Tremblade (lat 45.781741, long - 1.121910)
**F23**	Breeding program	Atlantic	Charente Maritime- La Tremblade (lat 45.781741, long - 1.121910)
**F28**	Breeding program	Atlantic	Charente Maritime- La Tremblade (lat 45.781741, long - 1.121910)
**F32**	Natural environment – non farming area	Mediterranean	Vidourle (lat 43.553906, long 4.095175)
**F33**	Natural environment – non farming area	Mediterranean	Vidourle (lat 43.553906, long 4.095175)
**F37**	Natural environment – non farming area	Mediterranean	Vidourle (lat 43.553906, long 4.095175)
**F42**	Natural environment – farming area	Mediterranean	Thau lagoon (lat 43.418736, long 3.622620)
**F44**	Natural environment – farming area	Mediterranean	Thau lagoon (lat 43.418736, long 3.622620)
**F48**	Natural environment – farming area	Mediterranean	Thau lagoon (lat 43.418736, long 3.622620)

Supplementary Table 2 : Primers used to validate RNA-seq data by RT-qPCR.

Gene name	ID in genbank or *C. gigas* genome	PCR efficiency	Sense and antisens primers (5'-3')
Cg-EF1	AB122066	1.97	GAGCGTGAACGTGGTATCAC
			ACAGCACAGTCAGCCTGTGA
Cg-RPL40	FP004478	2.02	AATCTTGCACCGTCATGCAG
			AATCAATCTCTGCTGATCTGG
Cg-RPS6	HS119070	2.01	CAGAAGTGCCAGCTGACAGTC
			AGAAGCAATCTCACACGGAC
Bactericidal permeability-increasing protein (BPI)	ACQ72939.1	2.04	ACGGTACAGAACGGATCTACG
			AATCGTGGCTGACATCGTAGC
Apoptosis Inducing factor1	CGI_10005410	2.06	AACAACCAGGTGGAACTAACC
			GACATTGCCTCCAGTTCAGC
Dicer-like protein 1	CGI_10020752	2	ACGTCGGTAGCAGAGGAAGG
			CTTCCTCCATCTTCTCACTGC
Ribonuclease 3	CGI_10005763	1.89	CCATTGTCTGTGATGACCTGG
			TCACAGAACTTGTAGCACACC
Epididymal secretory glutathione peroxidase-like	CGI_10025106	1.97	AGATCCGAGATGTCGTCTGG
			CAGACATGAGTTGCAGGATGG
60 kDa heat shock protein	CGI_10011081	1.9	AGGCTCTGGATAACGTCAAGG
			TCCAAAGTCTCCCTCGTTGC
Heat shock protein 83-like	CGI_10017621	2.02	TGGTTGGTCAGCAAACATGG
			TGTCTGCCTCAGCCTTATCC
Integrin beta-1-B-like	CGI_10009280	1.94	CACGACTACCCTCTGTAACGG
			TGTCCACGACTCAGACCTCC
Type I interferon receptor beta chain-associated protein	CGI_10017476	2.01	AGGGAAACTCCTCAGTCTGG
			GGGCTGCTGGAATGATTTCG
Piwi-like protein 1	CGI_10008757	1.87	TGGTGAGGAGTTGGTCAACG
			AGCATTCGTTGAGCTGAGGG
Stress-activated protein kinase JNK (JNK)	CGI_10020378	1.94	ATTCCCTCAGGACAGTCAGG
			CGTTGACTTCCTGCTCATCG
Superoxide dismutase (SOD)	CGI_10018833	1.91	TGGAACATGGCTGTGACACC
			TCTGTATGTCCGATGGTGAGC
TRAF-type zinc finger domain-containing 1	CGI_10022144	2.11	CTGCGAGTTCTGTGATGACC
			TTTGGCACGTGAAGCATTGG
complement component 3/4/5 (C3/4/5)	CGI_10014037	1.92	GTGTGCAGAATCATGGGATGC
			TGACAGTGGCTGAGAACACC
Krueppel-like factor 5	CGI_10000441	1.9	CCCAGGCTGCTCAACAATCA
			TGTGTTTTCGTATGTGGCGC
neuronal acetylcholine	CGI_10000478	2.04	GACGACCCTATCCCAACACC

receptor subunit alpha-6-like			ATCTCGCCGATCCTTTTCCC
metalloreductase STEAP4-like	CGI_10003627	2.1	AGATTGCCTGTCCAGTCACG
			AAACTTCATGGCTCCCTCCG
hypothetical protein	CGI_10019038	2.06	TGGCCACATTGTCCCTTCAG
			CGAAGGACCAGTTGAGGAGG
Complement C1q 2	CGI_10020815	2.08	TGGCCAACATGAACATGTCC
			TGCTCCCACTGTTGTACCAA
p38-α (MAPK14)	CGI_10004156	1.9	CACAGAAGCCCTGGCTCATC
			TGGTATAGTGAGTTCCATGTC
IRF-8	CGI_10003270	1.98	AGTCTGATCCAAATCTTGCAC
			GTCATCTGGGTATACTCCTC
TRAIL	CGI_10005109	1.99	GCAGAACGGCATGGAGTTTC
			CATAGGACTGGTAGAGGTC
STING	CGI_10003079	1.96	CTGCTATTGTCCGCCATC
			GAATGGGCGTGGCATACTC
ADAR2	CGI_10012998	2.05	CTCTGGGACTCACAGCAAC
			GTGTTTCCGTGTTCAATCATC
SOC	CGI_10019528	1.95	CAAGAGAGAATCTGTGGGAAC
			GCATCTTAGCACTAATTCTCTC
Interleukin Receptor	CGI_10003267	2.02	CAGAGGGAACCCAGGAATC
			CATCATTCGGTTGGCTGTGAC
Natterin-3	CGI_10014616	1.96	AGAATGTGGCGATCTTACACG
			ATTGGAAGCAAGCATCTGACG
Collagen alpha-5(VI) chain	CGI_10012008	2.03	AGCGAGCTGGGTCTTATTTCC
			TCTCCTTGAGGTCCCATTGG
BTG1	CGI_10015210	1.9	TCCATTCGACGTGTCCTACC
			ACATCATGGACATGGGTGAGG
major egg antigen-like isoform X1	CGI_10017582	2.02	TTCGGTGAGTGATGGGATGG
			ATAAAGCAGTGCACCTTGCC

Supplementary Table 3: Primers used for RT-qPCR of the S_{F14}, S_{F15}, R_{F23} and R_{F48} oyster families during the course of the 'natural' infection experiment.

Gene name	ID in genbank or *C. gigas* genome	PCR efficiency	Forward and reverse primers (5'-3')
Viral IAP (ORF106)	NC_005881	1.94	AGGAGGATTGTGGTCATTGC
			TCATCGTCAGAGTCGTCGTC
cGAS	CGI_10023476	1.92	TGGCTGAGAGAGCTATGCAA
			GCCTTTCTTCCTCTGGGACT
IRF	CGI_10021171	2.06	AAGAGGTGGAAGGCCAACTT
			TCGTTCGTTCTGCAGTCTTG
TNF	CGI_10005109	2.04	GCAGAACGGCATGGAGTTTC
			CATAGGACTGGTAGAGGTC
Viperin	CGI_10018396	1.92	TCAAGGACTTCTGCGAACG
			CCCGACATCTAGCAAAGAGC
Cg-IAP	CGI_10005393	2.08	TGGAACTAATGTGCGAGACG
			TCCATCTGCTGAATCAGTCC
SOC2	CGI_10019528	1.92	GGGGGACCACTAGTGTGAGG
			TCAAACGGGCATAGAAGTCC
Cg-BigDef2	JF703146	1.96	GGAGAGAAAATTCTGACCATGAC
			CATAGTTTATCCCCTCCGTC
Cg-PRP	JF766786	2.06	CACCATGTTCTCTCGGAGGA
			ATCTGCAATGTCAACCCTCTG
Cg-SOD	XM_011416094	1.97	AGAGGTGAATGCTACCAGG
			AGGCCAAGAATTCCGTCTG

Supplementary References

1 Petton, B. *et al.* Crassostrea gigas mortality in France: the usual suspect, a herpes virus, may not be the killer in this polymicrobial opportunistic disease. *Front Microbiol* **6**, 686, (2015).

2 Le Roux, F., Wegner, K. M. & Polz, M. F. Oysters and Vibrios as a Model for Disease Dynamics in Wild Animals. *Trends Microbiol* **24**, 568-580, (2016).

3 Davison, A. J. *et al.* A novel class of herpesvirus with bivalve hosts. *J Gen Virol* **86**, 41-53, (2005).
