# Supplementary material for: Immune-suppression by OsHV-1 viral infection causes fatal bacteraemia in Pacific oysters
Source: Nat Commun. 2018 Oct 11;9:4215. doi: 10.1038/s41467-018-06659-3 (PMC6182001; doi:10.1038/s41467-018-06659-3)
Supplement: Supplementary file 3 — Description of Additional Supplementary Files [file 41467_2018_6659_MOESM3_ESM.pdf]

## LEGENDS FOR SUPPLEMENTARY DATA

**Supplementary Data 1**: Absolute abundance of Operational Taxonomic Units (*ie* clusters) and their corresponding taxonomic affiliations for the susceptible $S_{F11}$ and the resistant $R_{F21}$ oysters. T0, T6, T12, T24, T48, T60 and T72 correspond to the different sampling times (in hour) during the kinetics of the 'natural' experimental infections. R1, R2, R3 correspond to the results of each replicate (Excel file).

**Supplementary Data 2**: Frequencies of bacterial taxa that are significantly modified over the course of the 'natural' experimental infection in the susceptible $S_{F11}$ and the resistant $R_{F21}$ oysters. T0, T6, T12, T24, T48, T60 and T72 correspond to the different sampling times (in hours) during the kinetics of the experimental infections. R1, R2, and R3 correspond to the results obtained for the different replicates (Excel file).

**Supplementary Data 3:** Log2 fold changes in differentially expressed genes (DESeq2) from the different GO categories of clusters 1 and 2 (Fig. 5b) at 6 and 12 h after the start of the 'natural' experimental infection in the susceptible $S_{F11}$ and in the resistant $R_{F21}$ oysters. *C. gigas* genes used for RT-qPCR in the susceptible $S_{F14}$ and $S_{F15}$, and in the resistant $R_{F23}$ and $R_{F48}$ oysters (Figure 9) are highlighted in purple (Excel file).

**Supplementary Data 4:** Gene ontology category enrichment for the susceptible $S_{F11}$ and the resistant $R_{F21}$ oysters at the different kinetic times (6, 12, 24, 48, 60 and 72 h) of the 'natural' experimental infection. GO enrichment analysis was performed using adaptive clustering and a rank-based statistical test. A category was considered enriched under a FDR of 1%. The ratios were calculated as the "number of genes that were significantly up-regulated in the category/total number of genes in the category". The ratio was positive if the category was up-regulated and negative if the category was down-regulated (Excel file).

**Supplementary Data 5:** Absolute abundance of Operational Taxonomic Units (*ie* clusters) and their corresponding taxonomic affiliations during the rationalized experimental infections by OsHV-1 and/or *V. crassostreae*. Donor oysters were injected with *V. crassostreae* and/or OsHV-1. Recipient oysters were injected with polyI:C (PIC) or sterile seawater (SW) before exposure to both donors (Vc+Os). Recipient oysters were exposed to both donors (Os+Vc) in the presence (Cm+) or absence (Cm-) of chloramphenicol in the tanks. Recipient oysters were exposed to only *V. crassostreae* (Vc) or OsHV-1 (Os) donors. For controls, recipients were exposed to non-injected donors. T0 and T72 correspond to the sampling times (in hours), and R1, R2, and R3 correspond to the results obtained for the different replicates (Excel file).

**Supplementary Data 6**: Frequencies of bacterial taxa that are significantly modified over the course of the rationalized experimental infections. Donor oysters were injected with *V. crassostreae* and/or OsHV-1. Recipient oysters were injected with polyI:C (PIC) or sterile seawater (SW) before exposition to both donors (Vc+Os). Recipient oysters were exposed to both donors (Os+Vc) in the presence (Cm+) or absence (Cm-) of chloramphenicol in the tanks. Recipient oysters were exposed to OsHV-1 (Os) donors. For controls, recipients were exposed to non-injected donors. No modification of the taxa occurred in recipients exposed to *V. crassostreae*-injected donors. T0 and T72 correspond to the sampling times (in hours), and R1, R2, and R3 correspond to the results obtained for the different replicates (Excel file).
